# The Synthesis Methodology and Characterization of Nanogold-Coated Fe_3_O_4_ Magnetic Nanoparticles

**DOI:** 10.3390/ma15093383

**Published:** 2022-05-09

**Authors:** Magdalena Kędzierska, Anna Drabczyk, Mateusz Jamroży, Sonia Kudłacik-Kramarczyk, Magdalena Głąb, Bożena Tyliszczak, Wojciech Bańkosz, Piotr Potemski

**Affiliations:** 1 Department of Chemotherapy, Medical University of Lodz, WWCOiT Copernicus Hospital, 90-001 Lodz, Poland; magdalena.kedzierska@umed.lodz.pl (M.K.); piotr.potemski@umed.lodz.pl (P.P.); 2 Department of Materials Science, Faculty of Materials Engineering and Physics, Cracow University of Technology, 37 Jana Pawła II Av., 31-864 Krakow, Poland; magdalena.glab@doktorant.pk.edu.pl (M.G.); bozena.tyliszczak@pk.edu.pl (B.T.); 3 Department of Automation and Robotics, Faculty of Electrical and Computer Engineering, Cracow University of Technology, 24 Warszawska St., 31-155 Krakow, Poland; wojciech.bankosz@doktorant.pk.edu.pl

**Keywords:** magnetic nanoparticles, gold nanoparticles, core-shell nanostructures, nanoparticles agglomeration, Arabic gum, sonication-assisted agglomerates disintegration, pro-inflammatory activity

## Abstract

Core-shell nanostructures are widely used in many fields, including medicine and the related areas. An example of such structures are nanogold-shelled Fe_3_O_4_ magnetic nanoparticles. Systems consisting of a magnetic core and a shell made from nanogold show unique optical and magnetic properties. Thus, it is essential to develop the methodology of their preparation. Here, we report the synthesis methodology of Fe_3_O_4_@Au developed so as to limit their agglomeration and increase their stability. For this purpose, the impact of the reaction environment was verified. The properties of the particles were characterized via UV-Vis spectrophotometry, dynamic light scattering (DLS), X-ray diffraction (XRD), and Scanning Electron Microscopy-Energy Dispersive X-ray analysis (SEM-EDS technique). Moreover, biological investigations, including determining the cytotoxicity of the particles towards murine fibroblasts and the pro-inflammatory activity were also performed. It was demonstrated that the application of an oil and water reaction environment leads to the preparation of the particles with lower polydispersity, whose agglomerates’ disintegration is 24 times faster than the disintegration of nanoparticle agglomerates formed as a result of the reaction performed in a water environment. Importantly, developed Fe_3_O_4_@Au nanoparticles showed no pro-inflammatory activity regardless of their concentration and the reaction environment applied during their synthesis and the viability of cell lines incubated for 24 h with the particle suspensions was at least 92.88%. Thus, the developed synthesis methodology of the particles as well as performed investigations confirmed a great application potential of developed materials for biomedical purposes.

## 1. Introduction

Magnetic nanoparticles belong to the group of nanomaterials whose properties change under the influence of the applied magnetic field. They appear as the magnetic fluid (defined also as ferrofluid), i.e., a colloidal suspension of nanoparticles in an inorganic or organic carrier liquid [1]. Metals such as iron, cobalt, manganese, or nickel, as well as their oxides or alloys, may also occur in the form of magnetic nanoparticles [2]. The magnetic nanoparticle shows the properties of a single magnetic domain, wherein this takes place under appropriate conditions: above the so-called blocking temperature. Such nanoparticles have a constant magnetic moment and, under the influence of a magnetic field, behave like an atom with paramagnetic properties. Chaotically ordered magnetic moments of unpaired electrons of such atoms align with the direction of the applied magnetic field, as a result of which the paramagnetic atom behaves like one large magnet [3].

One of the most commonly used magnetic nanoparticles are iron oxide nanoparticles. They show unique physicochemical properties, including magnetic and electrical ones. Importantly, there are several types of iron oxides including magnetite (Fe_3_O_4_/FeO · Fe_2_O_3_), maghemite (γ-Fe_2_O_3_), and hematite (α-Fe_2_O_3_) [4]. Magnetite shows the strongest magnetic properties among transition metal oxides. Iron oxide nanoparticles Fe_3_O_4_ with a core diameter of 5–15 nm and a hydrodynamic diameter of 20–150 nm are characterized by superparamagnetic properties [5]. Under the influence of an applied magnetic field, a single crystallite of such a superparamagnetic nanomaterial behaves like a single-domain particle with ordered magnetic moments. As a result, the applied magnetic field affects the magnetic moments of the crystallites and not individual atoms as it takes place, in the case of paramagnets. Moreover, when such structures are not subjected to the magnetic field, they do not exhibit magnetic properties [6].

The physicochemical properties of magnetic nanoparticles, among others, depend on the method of their synthesis. In addition, the parameters of the process and the reagents used have a significant impact on the shape of the nanoparticles, their size and crystallinity, as well as their dispersity (i.e., the particle size distribution in the post-reaction mixture) [7]. The mentioned factors are also related to the stability of the particles obtained and their tendency to form agglomerates. Therefore, it is extremely important to select an appropriate method of magnetic nanoparticle synthesis [8]. Among the methods leading to the preparation of this type of nanoparticles, such may be mentioned as biological, physical and chemical ones [9]. Due to the inability to control the size of the particles during physical processes [10], and the low efficiency of biological methods [11], the most commonly used are chemical methods including co-precipitation [12,13,14], thermal decomposition of organometallic compounds [15], the reverse micelle method [16,17,18], and the sol-gel method [19,20].

One problematic aspect of the use of magnetic nanoparticles is their high tendency to agglomerate [21]. This phenomenon is caused by the interactions between the hydrophobic surfaces of the nanoparticles. As a result, they form large clusters in which, additionally, magnetic attractions occur between individual nanoparticles. The decrease in the distance between nanomaterials with magnetic properties causes an increase in the magnetic interactions between them. This, in turn, leads to a more intensive agglomeration of the interacting nanoparticles [22]. Therefore, in order to prevent nanoparticle agglomeration, their surface is modified with an appropriately selected shell. This treatment is aimed at changing the surface properties of nanoparticles and, consequently, reducing the interactions between them. The surface of nanoparticles is modified so that their agglomeration is limited due to steric hindrances or electrostatic interactions. The former is achieved via the use of modifying agents such as polymeric materials of natural or synthetic origin. The second method is based on the selective adsorption of ions on the surface of nanoparticles [23,24].

Additionally, in order to prevent agglomeration of nanoparticles at the stage of their preparation, various types of surfactants (such as sodium oleate, sodium carboxymethylcellulose, or dodecylamine) are introduced into the reaction medium to increase the dispersibility of the resulting nanoparticles [25]. In the case of the target application of such nanostructures for biomedical purposes, it is important to select a surface modifier which shows biocompatibility, biodegradability, and stability in the environment of body fluids [26].

Considering problems with the agglomeration of magnetic nanoparticles, their low stability, or sometimes a low biocompatibility, new methods of their synthesis are still being developed [27]. Synthesis of magnetic nanoparticles for biomedical purposes requires the application of adequately selected parameters to ensure the possibility of obtaining nanoparticles with low distribution size, constant magnetic properties, good stability, and high biocompatibility [28]. Among the methods of obtaining magnetic nanoparticles, such methods as the chemical, physical, and biological ones may be mentioned. Among the physical ones, there are grinding, crushing in ball mills, vapor deposition, and lithographic methods involving the deposition of the desired shape on a previously prepared substrate, e.g., via UV radiation, X-rays, or an electron beam. Nonetheless, these methods are not used on a large scale due to the inability to control the particle size obtained during the above-mentioned processes. Biological methods involve a number of procedures using microbial enzymes or compounds of plant origin acting as reducing agents of metal ions. These methods are environmentally friendly (they do not require the use of toxic reagents) and the particles obtained usually show biocompatibility. However, the disadvantage of these procedures is their low efficiency and the high particle-size distribution. Due to the disadvantages of biological and physical methods, the method most often chosen for obtaining magnetic nanoparticles is the chemical one: the co-precipitation method, considered to be simple and highly effective. Moreover, this method is characterized by a high efficiency and the use of appropriate parameters ensures its high reproducibility [29,30,31,32].

In this work, the Massart synthesis (thus, the co-precipitation method) was used to prepare magnetic nanoparticles. The selection of the parameters of this synthesis allowed us to prepare nanoparticles with desired properties. The iron oxide magnetic nanoparticles were then modified to form a nanogold shell on their surfaces. Such a shell may limit the particles’ agglomeration and increase their application potential. Nanogold provides the so-called surface plasmon effect, which causes significant optical absorption in the visible and near infrared range [33]. Due to such optical properties of this nanomaterial, the Fe_3_O_4_@Au particles obtained may also be applied in photothermal therapies and in imaging [34]. Systems consisting of a magnetic core and a shell made from nanogold show unique optical and magnetic properties. Thus, it is essential to develop a methodology to be used to obtain them [35]. Nonetheless, the majority of the methods used for their preparation requires the use of environmentally hazardous organic reagents. For example, Alonso-Cristobal et al. presented a synthesis methodology using, among others, hexane 1-octadecene, oleylamine, and diphenyl ether [36]. On the other hand, the method proposed by Felix et al. requires the use of acetylacetonate, 1,2-hexadecanediol, oleylamine, oleic acid, and 1-octadecane [37]. Next, Caro et al. performed synthesis using N,N-dimethylformamide (DMF) [38], while Wang used phenyl ether and 1,2-hexadecanediol as reagents [39]. The synthesis methodology of Fe_3_O_4_@Au proposed in this work does not require the use of organic solvents. Furthermore, the presented method may be performed in the same conditions (temperature, constant stirring, and an inert gas atmosphere) as the synthesis leading to the preparation of Fe_3_O_4_ nanoparticles. This, in turn, is of great convenience and reduces the need for a variety of reaction systems. Moreover, the particles obtained were characterized in detail while their physicochemical analysis supported with biological investigations presented in this work confirmed a great application potential of developed materials.

## 2. Materials and Methods

### 2.1. Materials

Iron (II) chloride hexahydrate (97%), iron (III) chloride tetrahydrate (98%), hydrochloric acid (36.5–38.0%), and hydroxylamine hydrochloride (98%) were purchased from Sigma Aldrich (Saint Louis, MO, USA). Sodium hydroxide (pure p.a., 98.8%), tetrachloroauric (III) acid (99.995% trace metals basis), and Arabic gum (powder) were bought from Avantor Performance Materials Poland S.A. (Gliwice, Poland). Essential oil used as a component of the reaction environment of Fe_3_O_4_@Au nanoparticles was bought in AromaLab (Warsaw, Poland).

### 2.2. Synthesis of Fe_3_O_4_ Nanoparticles via Massart Synthesis

Iron oxide particles were obtained via Massart synthesis, i.e., the chemical co-precipitation of iron ions in an alkaline environment [40,41]. This procedure was described in detail in our previous article [42]. After the reaction, the obtained precipitate of magnetic nanoparticles was washed with 0.1 M sodium citrate solution. Next, the suspension was treated with a magnet to verify the behavior of the particles obtained under the influence of an external magnetic field. Finally, the suspension obtained was lyophilized and subjected to studies aimed at characterizing the particles’ crystallinity and surface morphology.

### 2.3. Synthesis of Fe_3_O_4_@Au Nanoparticles

**Figure 1:**
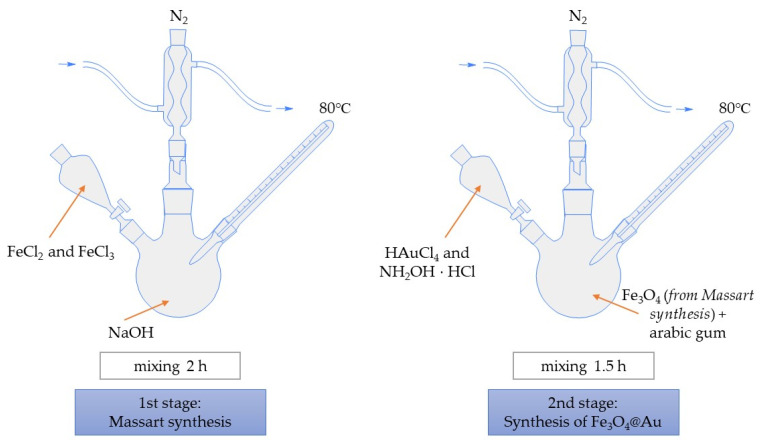
The scheme of Fe_3_O_4_@Au synthesis.

At the beginning, 7.5 mL of Fe_3_O_4_ nanoparticles suspension obtained as a result of the Massart synthesis was added to 3% aqueous solution of Arabic gum. Such a mixture was then heated to 80 °C. When this temperature was achieved, 0.2 M tetrachloroauric (III) acid (3.75 mL) acted as a source of gold ions (Au^3+^) and 0.1 M hydroxylamine hydrochloride (1.50 mL) acted as a reducing agent (introduced into the mixture to reduce Au^3+^ ions into Au^0^). Such a reaction was maintained for 1 h at 80 °C. Importantly, Fe_3_O_4_@Au nanoparticles synthesis was carried out with constant stirring and in an inert gas (argon) atmosphere. In Figure 1, the scheme of the synthesis of Fe_3_O_4_@Au nanoparticles is presented.

Furthermore, the synthesis of Fe_3_O_4_@Au particles was performed in two reaction environments. The first variant included the use of 140 mL of 3% solution of Arabic gum in distilled water (known as the water environment, W environment). The second reaction environment included the combination of the aqueous solution of Arabic gum with an essential oil in a 1.4:1.0 volume ratio (known as the oil and water environment, O/W environment). Next, the particles obtained as a result of the application of various reaction environments were characterized to verify the impact of the reaction conditions on such features of the resulting particles as their size, stability, or biological properties.

#### Analysis of the Impact of the Reaction Parameters on the Efficiency of Fe_3_O_4_@Au Nanoparticles Synthesis

The next step of the research included verifying the impact of the temperature applied as well as the molar ratio of tetrachloroauric (III) acid and the reducing agent on the efficiency of Fe_3_O_4_@Au particles synthesis. For this purpose, various synthesis conditions were applied:

- the molar ratio HAuCl_4_:NH_2_OH·HCl—4:3, temperature—25 °C (variant 1),
- the molar ratio HAuCl_4_:NH_2_OH·HCl—4:3, temperature—80 °C (variant 2),
- the molar ratio HAuCl_4_:NH_2_OH·HCl—4:2, temperature—80 °C (variant 3).

After such reactions, the suspensions obtained were analyzed via UV-Vis spectrophotometry to verify the formation of Fe_3_O_4_@Au nanoparticles.

### 2.4. Analysis of Particles’ Crystallinity

Crystallinity of the particles obtained as a result of Massart synthesis was characterized by the X-ray diffraction (XRD) technique. The study was performed by means of a Bruker D2 Phaser diffractometer. All analyses were conducted in the reflection mode (kCu = 1.54 Å), at ambient temperature while the measurement range was 5–40°, respectively.

### 2.5. Analysis of the Particles Size via Dynamic Light Scattering (DLS)

One of the most significant parameters of the particles obtained—both Fe_3_O_4_ as well as Fe_3_O_4_@Au particles—is their size, which was determined by means of DLS technique. For this purpose, a Zetasizer Nano ZS Malvern was applied. All measurements were conducted at ambient temperature (25 °C).

### 2.6. Investigations on the Optical Properties of Fe_3_O_4_@Au Particles

UV-Vis spectrophotometry was used to verify the formation of the shell made from nanogold on the surface of iron oxide magnetic nanoparticles. The analysis was performed using a ThermoScientific Evolution 220 UV-Vis spectrometer. The study was performed at ambient temperature and within the wavelength range 300–700 nm.

### 2.7. Ultrasound-Assisted Disintegration of the Particles Agglomerates

In order to disintegrate the formed particle agglomerates, the sonication process was proposed. This is based on the application of ultrasounds. The procedure was performed using an Omnic Sonic Ruptor ultrasound homogenizer (power 40%, pulsation 50%).

### 2.8. Characterization of the Particles’ Surface Morphology Supported with the Chemical Composition Analysis Using Scanning Electron Microscope (SEM) with Energy Dispersive X-ray Spectroscopy (SEM-EDS Method)

Surface morphology of the particles as well as their chemical compositions were defined via a Scanning Electron Microscope equipped with EDS detector (voltage: 10 kV). The study was conducted by means of a Helios NanoLab H50HP FEI microscope while an accelerating voltage 5 kV was applied to obtain images.

### 2.9. Analysis of the Particles via Transmission Electron Microscopy (TEM)

The surface morphology of the particles obtained was also investigated via transmission electron microscopy. The JEOL JEM1200 (JEOL USA Inc., Peabody, MA, USA) transmission electron microscope was applied while the TEM images were recorded at the accelerating tension 120 kV.

### 2.10. Biological Analysis of the Obtained Particles

#### 2.10.1. Analysis of Antimicrobial Properties of Prepared Particle Suspensions

This study involved determining the antimicrobial activity of obtained particle suspensions. For this purpose, two parameters were verified, i.e., the minimum concentration inhibiting microbial growth (MIC) and the minimum bactericidal concentration (MBC) against pathogens that mainly cause the bacterial infections of soft tissues and skin. In order to determine these parameters, the broth microdilution method recommended by the Clinical and Laboratory Standards Institute (CLSI; standard M07-A9 [43]) was applied. Analyses were performed using *Staphylococcus aureus* ATCC^®^25923™ and *Staphylococcus epidermidis* ATCC^®^12228™ bacterial strains. These strains were plated on Műeller–Hinton agar (MHA) microbial medium and cultured for 24 h at 37 °C.

Subsequently, the bacterial strain suspensions with a density of 10^8^ CFU/mL in a 0.85% NaCl solution were prepared, then diluted to a density of 5 × 10^5^ CFU/mL in Műeller–Hinton (MHB) medium. The nanoparticle suspensions were also prepared in this medium. Subsequently, 100 µL of the nanoparticle suspensions were introduced into the wells of a 96-well plate, then 100 µL of the microorganism suspension (with a density of 5 × 10^5^ CFU/mL) was added into each well. Importantly, the analysis also included the control of the growth of microorganisms (K^+^, well containing 100 µL of the microorganism suspension and 100 µL of the culture medium) and the sterility control of the culture medium (K^−^, well containing 200 µL of the culture medium). All resulting suspensions were incubated for 24 h at 37 °C, after which the MIC and MBC values were determined via the visual reading. The minimum concentration inhibiting the growth of microorganisms was considered to be the concentration of nanoparticle suspension in which the substrate was not turbid (which would indicate the growth of microorganisms).

#### 2.10.2. Analysis of Cytotoxicity of the Particles via MTT Reduction Assay

Considering the application of developed materials for biomedical purposes, it is important to determine their cytotoxicity towards selected cell lines. For this purpose, a MTT reduction assay using an L929 murine fibroblasts cell line was performed. This is an enzymatic test based on determining the enzymatic activity of living cells. In order to verify such an activity, the soluble 3-(4,5-dimethylthiazol-2-yl)-2,5-diphenyl tetrazolium bromide (defined as MTT reagent) is introduced into the analyzed environment. This compound is then converted into insoluble formazan wherein the reaction proceeds in the presence of a mitochondrial dehydrogenase, i.e., the enzyme secreted by the living cells. Formazan crystals are then dissolved in DMSO (or isopropanol), where the intensity of the color of the formed solution corresponds to the amount of formed formazan crystals and to the amount of the enzyme secreted by tested cells (so, it also corresponds to the viability of these cells). The solution formed is evaluated spectrophotometrically at 570 nm and provides information on the amount of properly functioning living cells (and at the same time on their viability). The procedure of MTT reduction assay as well as the L929 murine fibroblasts culture have been previously described in [44]. The study was performed both for nanoparticle suspension obtained in the water environment and in the oil and water (O/W) environment to verify the impact of the reaction environment on the cytotoxicity of obtained suspensions.

#### 2.10.3. Evaluation of the Pro-Inflammatory Activity of the Particles

The proinflammatory (immunostimulatory) activity of nanoparticle suspensions was verified using THP1XBlueTM cells (InvivoGen, San Diego, CA, USA). This is a genetically modified human monocytic macrophage cell line. The analysis involved determining the amount of the germline alkaline phosphatase (SEAP), which is released by tested cells into the tested environment as a result of the cellular response to stress or to the presence of pathogens. SEAP forms a complex with a QuantiBlue^TM^ reagent and such a complex may be determined spectrophotometrically. The whole procedure of determining the pro-inflammatory activity as well as the cell line culture has been described in [45]. The analysis was conducted both for nanoparticle suspension obtained in the water environment and in the oil and water (O/W) environment to check the potential impact of the reaction environment applied on the pro-inflammatory activity of nanoparticle suspensions.

## 3. Results and Discussion

### 3.1. Synthesis of Iron Oxide Magnetic Nanoparticles

**Figure 2:**
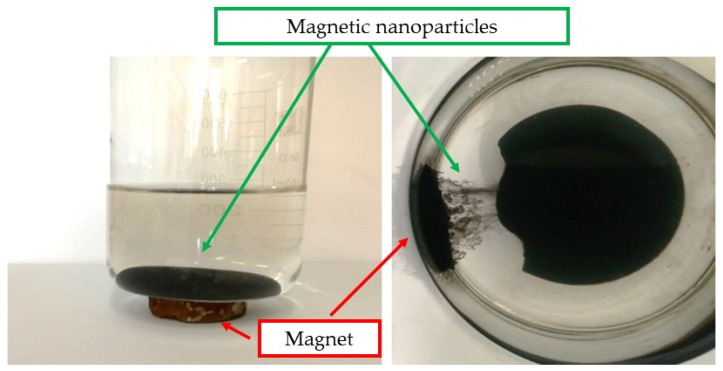
Images showing the behavior of the nanoparticles under the influence of the magnetic field applied.

In Figure 2 the behavior of the particles under the influence of the magnetic field is presented.

In Figure 2, the migration of the obtained Fe_3_O_4_ magnetic nanoparticles towards the applied magnetic field (magnet) has been presented. Figure 2 (on the left) shows the accumulation of nanomaterials at the bottom of a laboratory vessel to which the magnet is applied. Such behavior of nanoparticles confirms their magnetic properties and their ability to direct their movement using an external stimulus, i.e., a magnetic field. Nanoparticles migrate to the place where the magnet is placed, which is presented in Figure 2 (on the right). The magnet was placed on the outside of the wall of the laboratory vessel and, as a result, the migration of nanoparticles accumulated at the bottom of the vessel towards its wall is observed.

### 3.2. Results of the Analysis of the Surface Morphology via SEM Technique

**Figure 3:**
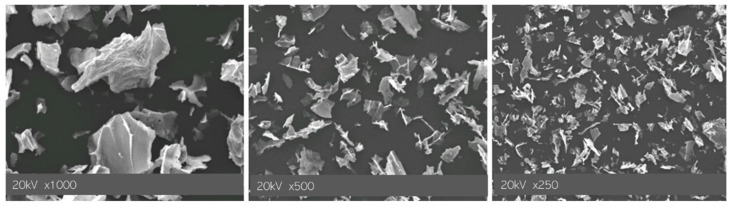
SEM images of Fe_3_O_4_ nanoparticles.

In Figure 3 images of particles obtained via SEM technique are presented.

In Figure 3, the morphology of prepared Fe_3_O_4_ nanoparticles, obtained as a result of Massart synthesis, may be observed. The visible structures do not indicate the preparation of nanoparticles having a spherical shape. Nonetheless, a similar observation was made in other literature reports [46]. Prepared nanomaterials have a structure of flakes. This may be caused by the fact that the synthesis of these particles was conducted using the Arabic gum as the stabilizing agent.

### 3.3. Analysis of Crystallinity via XRD Technique

**Figure 4:**
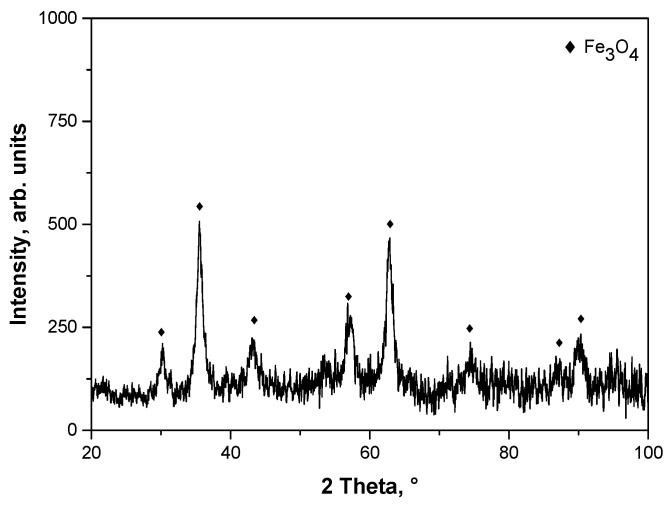
XRD pattern of magnetic nanoparticles washed with 0.1 M sodium citrate solution.

In Figure 4, XRD diffractogram of Fe_3_O_4_ nanoparticles is presented while, in Table 1, parameters determining their crystallinity are shown.

In Table 1, the parameters determined based on performed XRD analysis are summarized.

On the diffraction pattern in Figure 4, the presence of reflections at 2θ = 30.08°, 35.44°, 43.21°, 58.02°, or 62.45° may be observed. Such peaks are characteristic for Fe_3_O_4_ iron oxide nanoparticle crystals with the cubic structure of an inverted spinel (Fe_3_O_4_, ICDD 00-001-1111) [47]. Importantly, only the peaks that are characteristic of magnetite nanoparticles were observed on the obtained XRD diffraction pattern. No reflections attributable to the maghemite were noticed. This proved the phase purity of the obtained material.

### 3.4. Evaluation of the Impact of the Reaction Environment Applied on the Size and Agglomeration of Fe_3_O_4_@Au Particles

**Figure 5:**
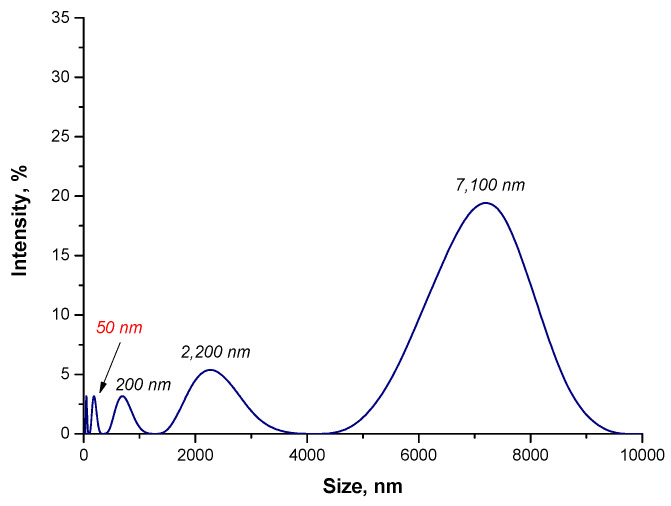
The size analysis of Fe_3_O_4_@Au particles obtained in the water environment.

In order to verify the impact of the reaction environment both on the size of the particles obtained and their tendency to form agglomerates as well as on the disintegration of agglomerates formed, DLS analysis was performed. In Figure 5, the results of the size analysis of the particles obtained during the reaction performed in the water environment are presented.

**Figure 6:**
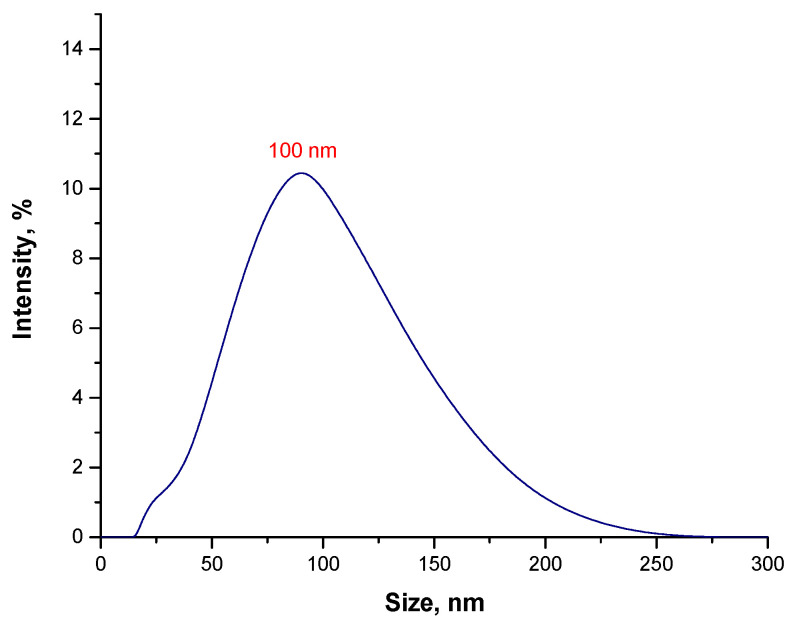
The size analysis of Fe_3_O_4_@Au particles obtained in the water environment and subjected to 6 h sonication.

Based on the DLS analysis, it may be reported that the particles with a large size distribution are present in the tested suspension. Importantly, the particles with a size of 50 nm—so, nanoparticles—were noticed. However, the micrometric particles with a size of 7100 nm (with an intensity of 20%) and 2200 nm (with an intensity of approximately 5%) may also be observed. Moreover, the particles with a size of 200 nm were also observed. They are probably agglomerates of magnetic nanoparticles. Thus, this may indicate that Fe_3_O_4_ particles form agglomerates. In order to disintegrate them, the sonication process was applied. After this process, the size of the particles was verified again via the DLS technique. Results of this analysis are presented below in Figure 6.

As a result of the sonication process, all agglomerates of particles disintegrated. Importantly, in the analyzed suspension, the presence of nanoparticles was observed. Thus, it was found that the sonication process turned out to be effective in breaking up the nanoparticle agglomerates.

The sonication process involved the use of high-frequency sound waves (ultrasounds). During the sonication of liquids, sound waves propagate in the liquid medium. This, in turn, generates alternating cycles of low and high pressure. The speed at which each cycle occurs depends strictly on the ultrasound. During low pressure cycles, small vacuum bubbles are formed, which is generated by high-intensity sound waves. When the resulting bubbles reach a size (volume) at which it is no longer possible to absorb energy, they are rapidly collapsed during the high pressure (compression) cycle. The phenomenon of collapsing bubbles is accompanied by a local rapid increase in temperature and pressure. In addition, the bubble implosion also causes turbulence of the liquid. Therefore, as a result of the use of sonication towards the suspension with nanoparticle aggregates, cavitation of bubbles in the vicinity of such an agglomerate reduces the interactions between the agglomerated particles. This, in turn, leads to their disintegration [48].

Nonetheless, in the case of the tested suspension, 6 h sonication was necessary to disintegrate all micrometric agglomerates. Thus, it was time-consuming and energy-consuming.

**Figure 7:**
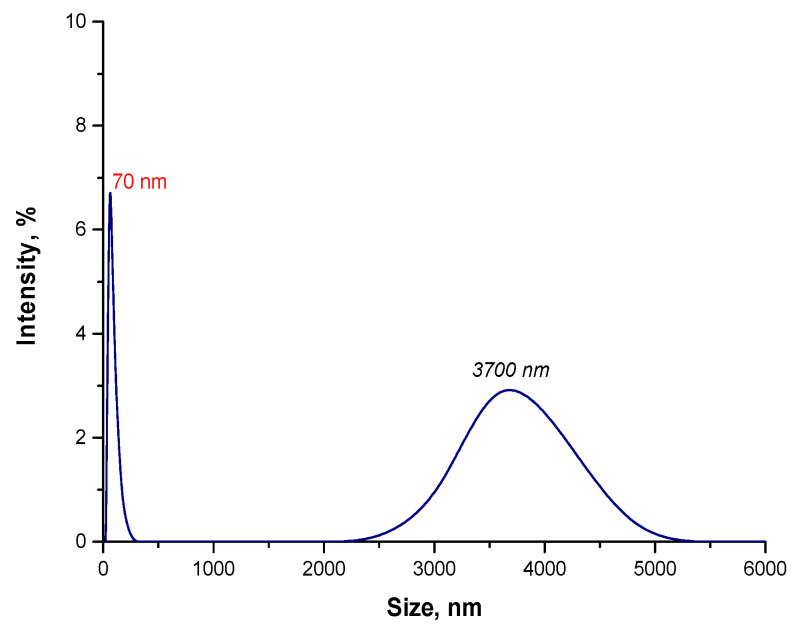
The size analysis of Fe_3_O_4_@Au particles obtained in the oil and water environment.

In the next step, the size analysis of the obtained particles as a result of the reaction performed in an oil and water environment was conducted. Results of the study are presented below in Figure 7.

**Figure 8:**
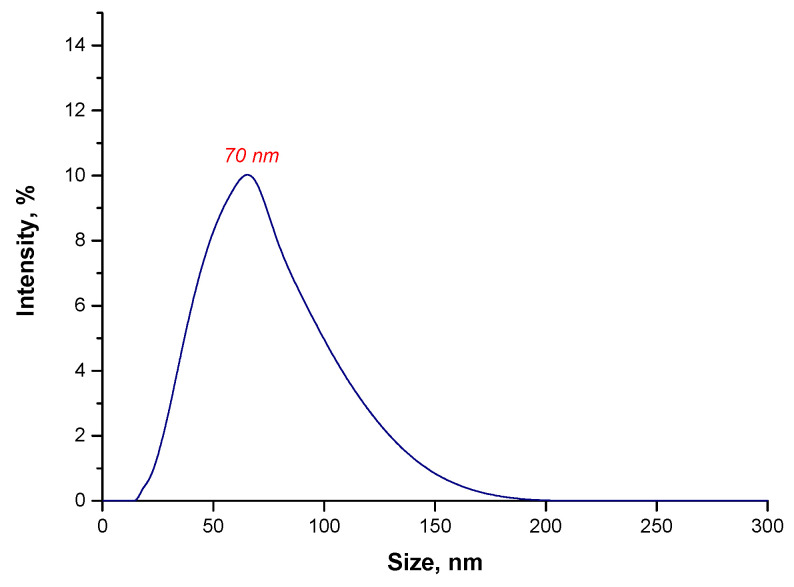
The size analysis of Fe_3_O_4_@Au particles obtained in the oil and water environment after 15 min sonication.

In the suspension, as a result of the synthesis of Fe_3_O_4_@Au particles in the oil and water environment, the particles with a size of 70 nm were obtained. However, agglomerates of particles with a size of approximately 3700 nm were also noticed. Thus, it may be concluded that the reaction environment affected the size of the obtained particles. In the oil and water environment, the oil droplets dispersed in the resulting O/W emulsion create a physical barrier between nanoparticles in the suspension; thus, limiting interactions between nanoparticles, which might lead to the formation of agglomerates. Additionally, Arabic gum present in the suspension causes the occurrence of steric stabilization. This biopolymer adsorbs on the surface of magnetic nanoparticles, increasing the distance between them. Due to the presence of agglomerates with a size of 3700 nm in the tested suspension, the sonication process was used at a later stage in order to disintegrate them. After ultrasound treatment, the suspension was re-analyzed using the DLS method and the obtained results are presented below in Figure 8.

As it may be observed in Figure 8, all particle agglomerates disintegrated as a result of a 15 min sonication process. In the tested suspension, the particles with a size of approximately 70 nm may only be observed. Particles of the desired size, i.e., nanoparticles, are obtained after a 15 min sonication of the treatment of this suspension, using ultrasounds while, in the case of the particle suspension obtained during the reaction performed in the water environment, it was necessary to apply ultrasounds for 6 h. Thus, it was demonstrated that from an economical viewpoint, the synthesis conducted in the oil and water environment is the most favorable.

### 3.5. Evaluation of Optical Properties of Obtained Particles via UV-Vis Spectrophotometry

**Figure 9:**
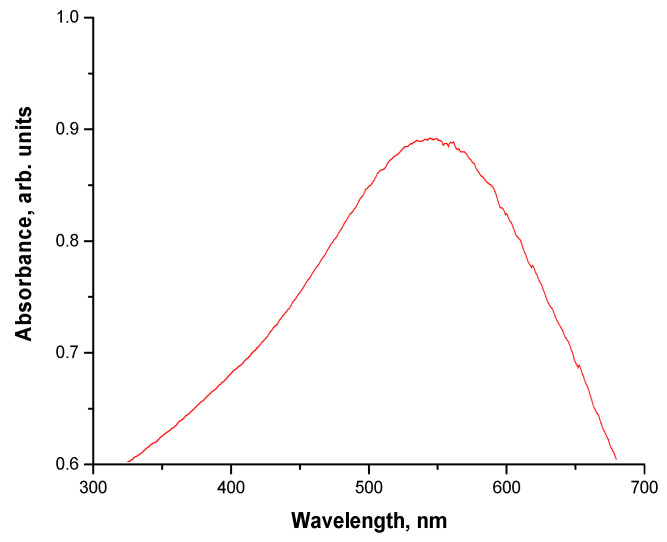
UV-Vis spectrum of Fe_3_O_4_@Au particles obtained in the oil and water environment.

The particle suspension was next analyzed using UV-Vis spectrophotometry to verify whether the nanogold shell was formed on the surface of Fe_3_O_4_ nanoparticles. Obtained UV-Vis spectrum is presented below in Figure 9.

The absorption band with a maximum absorbance at a wavelength of approx. 540 nm may be observed on the obtained UV-Vis spectrum. According to the literature, a maximum absorbance characteristic for gold nanoparticles is within the range 510–530 nm [49]. The maximum observed is outside this range. Nonetheless, such a slight shift probably results from the presence of magnetic nanoparticles, while the mentioned range concerns pure gold nanoparticles. Similar results concerning the shift of the maximum absorbance characteristic for nanogold were observed in [50,51,52]. Thus, based on the results of the UV-Vis study, it may be concluded that, as a result of performed synthesis, nanoparticles with a magnetic core and with a shell made from nanogold were prepared.

### 3.6. Studies on the Impact of the Reaction Parameters on the Efficiency of Fe_3_O_4_@Au Nanoparticles Synthesis

**Figure 10:**
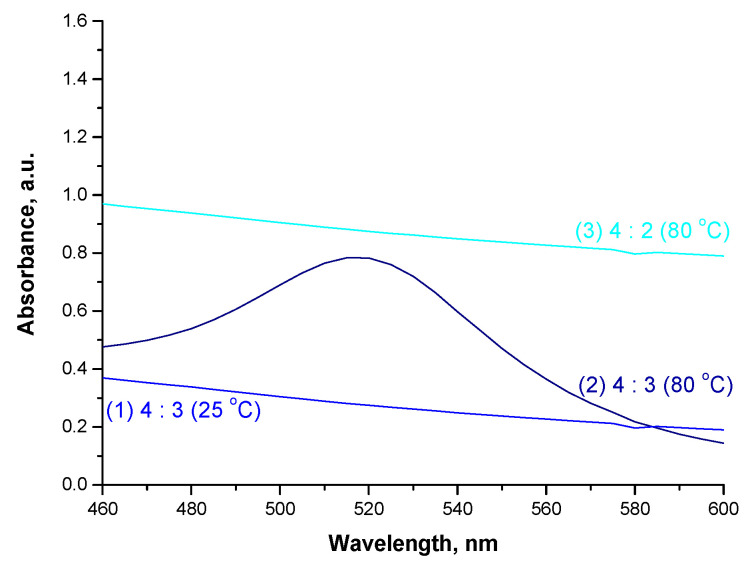
UV-Vis spectra of suspensions obtained under various Fe_3_O_4_@Au nanoparticle synthesis conditions.

In Figure 10, UV-Vis spectra of the suspensions obtained under various conditions of Fe_3_O_4_@Au nanoparticles synthesis are presented.

The absorption band with the maximum absorbance at a wavelength of approx. 520 nm, indicating the presence of nanogold, was observed only in the case of UV-Vis spectrum of suspension obtained under the following conditions: the reaction temperature at 80 °C and the molar ratio of chloroauric acid (provides gold ions) to reducing agent = 4:3. The molar ratio of the reagents applied corresponds to their stoichiometric ratio in the chemical reaction of the reduction of Au^3+^ to Au^0^. During this reduction the following reaction (1) takes place:

(1) $$3{\mathrm{NH}}_{2}\mathrm{OH}\cdot \mathrm{HCl}+4{\mathrm{HAuCl}}_{4}+3H_{2}O\;\to 3{\mathrm{HNO}}_{2}+4{\mathrm{Au}}^{0}+19\mathrm{HCl}$$

During the investigations various molar ratios of the chloroauric acid and hydroxylamine hydrochloride were verified as well as the efficiency of the reduction at ambient temperature. It was proved that the synthesis of Fe_3_O_4_@Au nanoparticles (thus, structures with a magnetic core and a shell made from nanogold) is successful when it is performed with a stoichiometric ratio of the mentioned reagents and at an elevated temperature (80 °C).

### 3.7. SEM-EDS Analysis of Fe_3_O_4_@Au Nanoparticles

**Figure 11:**
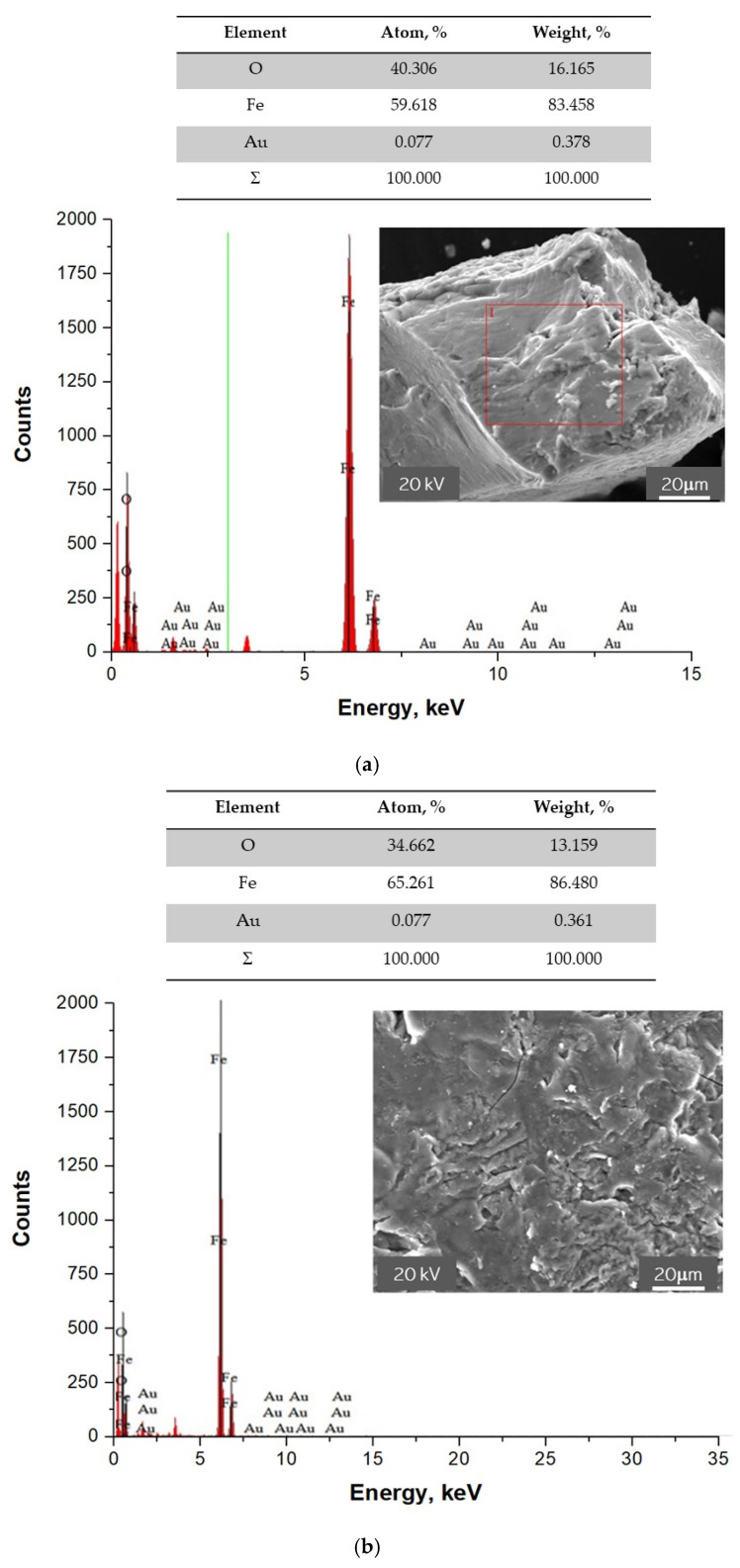
SEM images of Fe_3_O_4_@Au nanoparticles supported with EDS analysis of two points (**a**,**b**).

In Figure 11, images presenting the surface morphology of the particles obtained supported with their point analysis of the elemental composition are shown.

Based on the performed point elemental analysis of the particles obtained, the presence of chemical elements, such as iron, oxygen, and gold in the tested material was found. Thus, the study allowed us to confirm that, as a result of the synthesis, Fe_3_O_4_@Au nanoparticles consisting of a magnetic core from iron oxide nanoparticles and a shell made from gold nanoparticles were obtained.

### 3.8. Analysis of the Particles via TEM Technique

**Figure 12:**
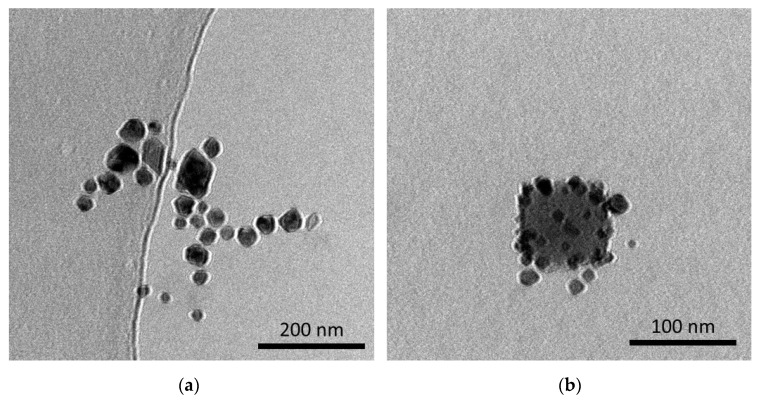
TEM images of the Fe_3_O_4_ particles after Massart synthesis (**a**) and Fe_3_O_4_@Au nanoparticles (**b**).

In Figure 12, TEM images of the particles are presented.

TEM technique allows us to visually verify the difference between the Fe_3_O_4_ particles obtained via Massart synthesis and Fe_3_O_4_@Au nanoparticles. In Figure 12a, the agglomerate of iron oxide nanoparticles may be observed. The image obtained confirmed that these particles occur close to each other, which results in the formation of their agglomerates. On the other hand, in Figure 12b iron oxide nanoparticle coated with smaller nanoparticles may be noticed. This, in turn, confirmed the formation of core-shell structures.

### 3.9. Studies on the Antimicrobial Activity of the Particle Suspension

In Table 2, results of the study on antimicrobial properties of the particle suspensions are presented.

Based on the results of the analysis, it was demonstrated that Fe_3_O_4_@Au nanoparticles did not show antibacterial properties. The only exception were the nanoparticles that were prepared in an oil and water reaction environment, which show antibacterial activity against Staphylococcus aureus ATCC 25923. Nonetheless, investigated nanomaterials were developed for application as drug carriers in anti-cancer therapies. Thus, in the case of such an application, the antimicrobial properties are not required. On the other hand, the possibility of modifying the surface of such materials allows for, e.g., the preparation of Fe_3_O_4_@Ag nanoparticles (with a shell made from silver nanoparticles, which are characterized by antimicrobial properties [53]). Such structures have been described by us in [42].

### 3.10. Cytotoxicity Assessment via MTT Reduction Assay

**Figure 13:**
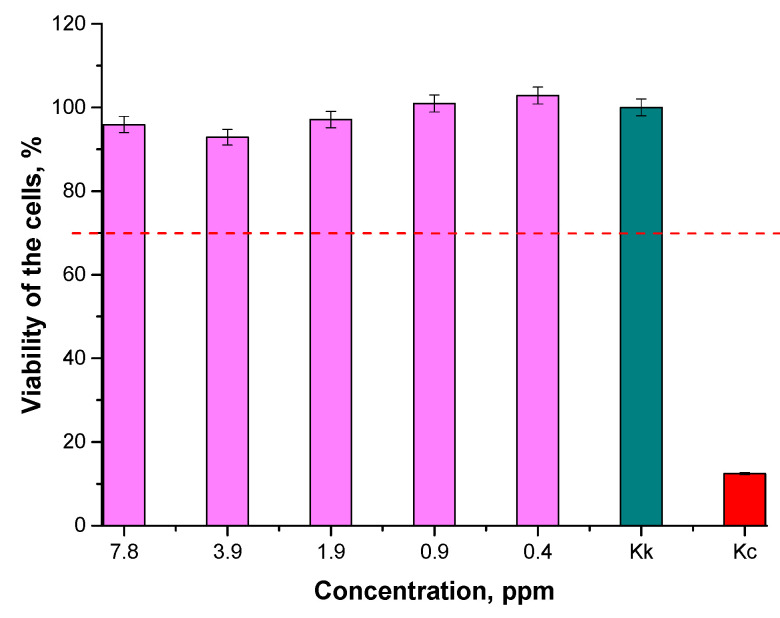
Results of MTT reduction assay of Fe_3_O_4_@Au nanoparticle suspensions prepared in the water environment (the cell viability over 70%—this has been marked via a red dotted line—indicates non-cytotoxicity of tested materials).

**Figure 14:**
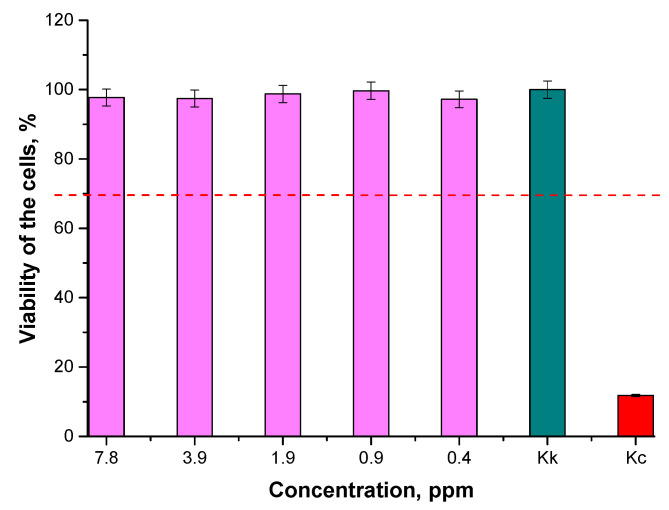
Results of MTT reduction assay of Fe_3_O_4_@Au nanoparticle suspensions prepared in the oil and water environment (the cell viability over 70%—this has been marked via a red dotted line—indicates non-cytotoxicity of tested materials).

In Figure 13 and Figure 14, results of MTT assays of the particle suspensions prepared in various environments are shown.

According to the ISO standard (EN ISO 10993-5: 2009 Biological evaluation of medical devices—Part 5: In vitro cytotoxicity studies), a biomaterial is not considered to be cytotoxic when the survival rate of cell lines incubated in its presence for 24 h is over 70% [54]. Thus, based on the results of the performed MTT reduction assay, it may be reported that the Fe_3_O_4_@Au nanoparticle suspensions at concentrations of 0.4, 0.9, 1.9, 3.9, and 7.8 ppm were not cytotoxic towards L929 murine fibroblasts. Therefore, it may be concluded that the obtained materials may be considered for application as potential drug carriers, because they do not cause cytotoxic effects towards the tested cell lines.

### 3.11. Evaluation of Pro-Inflammatory Activity

**Figure 15:**
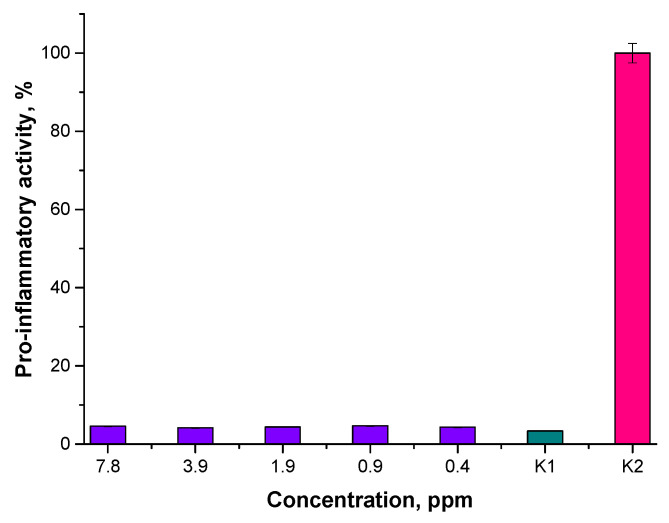
Analysis of the pro-inflammatory activity of Fe_3_O_4_@Au nanoparticle suspensions prepared in the water environment.

**Figure 16:**
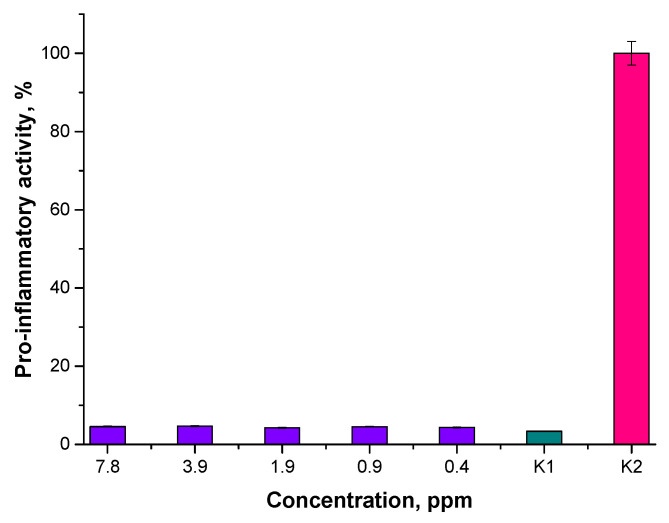
Analysis of the pro-inflammatory activity of Fe_3_O_4_@Au nanoparticle suspensions prepared in the oil and water environment.

Results of the studies on pro-inflammatory properties are shown below in Figure 15 and Figure 16.

Performed analyses allowed us to conclude that the tested nanoparticle suspensions, regardless of their concentration or the reaction environment applied, did not exhibit pro-inflammatory activity. The results obtained for the tested materials are similar to the results of the study carried out with the use of unstimulated monocytes in the culture medium (K1 control).

## 4. Conclusions

- Massart synthesis obtained Fe_3_O_4_ iron oxide nanoparticle crystals with the cubic structure of inverted spinel. Moreover, the proposed synthesis conditions obtained Fe_3_O_4_ with a phase purity, which was confirmed via XRD diffraction pattern.
- The applied reaction environment had a significant impact on the size of magnetic nanoparticles and the stability of their suspensions. It was proved that the reaction environment providing the preparation of materials with a small particle size distribution was the oil and water environment. Oil droplets dispersed in distilled water cause additional stabilization and limit the agglomeration of magnetic nanoparticles.
- The sonication process is an effective method of disintegrating nanoparticle agglomerates. Regardless of the type of the reaction environment applied, this process enables the disintegration of formed nanoparticle agglomerates. However, in the case of the water environment, complete disintegration of the agglomerates (i.e., obtaining a suspension containing only nano-sized particles) was achieved after a few hours. In turn, in the case of an oil and water environment, 15 min of sonication resulted in obtaining a monodisperse suspension of nanoparticles.
- Iron oxide nanoparticles with a shell from gold nanoparticles did not show antimicrobial properties. Moreover, regardless of the reaction environment applied during the synthesis, these nanomaterials did not exhibit cytotoxic properties towards the L929 murine fibroblast cells and pro-inflammatory activity against the THP1XBlueTM cell line.
- The obtained materials constitute a promising material for modification with the use of the drug substances, and may be used as their potential carrier. Importantly, due to their magnetic properties, the drug could be delivered to the specific site in the patient body by applying an external magnetic field.

## Figures and Tables

**Table 1:** Parameters determining the crystallinity of obtained particles.

Chemical Compound(ICDD)	Space Group	Network Type	Pattern Network Parameter (ICDD), Å	Calculated Network Parameter, Å	Network Deformation, %	Crystallinity Size, nm
Fe_3_O_4_ (00-001-1111)	Fd-3m	cubic	a = 8.374	a = 8.368	−0.39	10

**Table 2:** Antimicrobial activity of analyzed particle suspensions.

Bacterial Strain	*S. aureus* ATCC 25923		*S. epidermidis* ATCC 12228	
Sample	MIC (µL/mL)	MBC (µL/mL)	MIC (µL/mL)	MBC (µL/mL)
Fe_3_O_4_@Au_W	>100	>100	>100	>100
Fe_3_O_4_@Au_O/W	50	>100	>100	>100

## Data Availability

Data sharing is not applicable for this article.
